# Supplementary figures and images for: Irisin ameliorates myocardial ischemia-reperfusion injury by modulating gut microbiota and intestinal permeability in rats
Source: PLoS One. 2023 Sep 1;18(9):e0291022. doi: 10.1371/journal.pone.0291022 (PMC10473488; doi:10.1371/journal.pone.0291022)

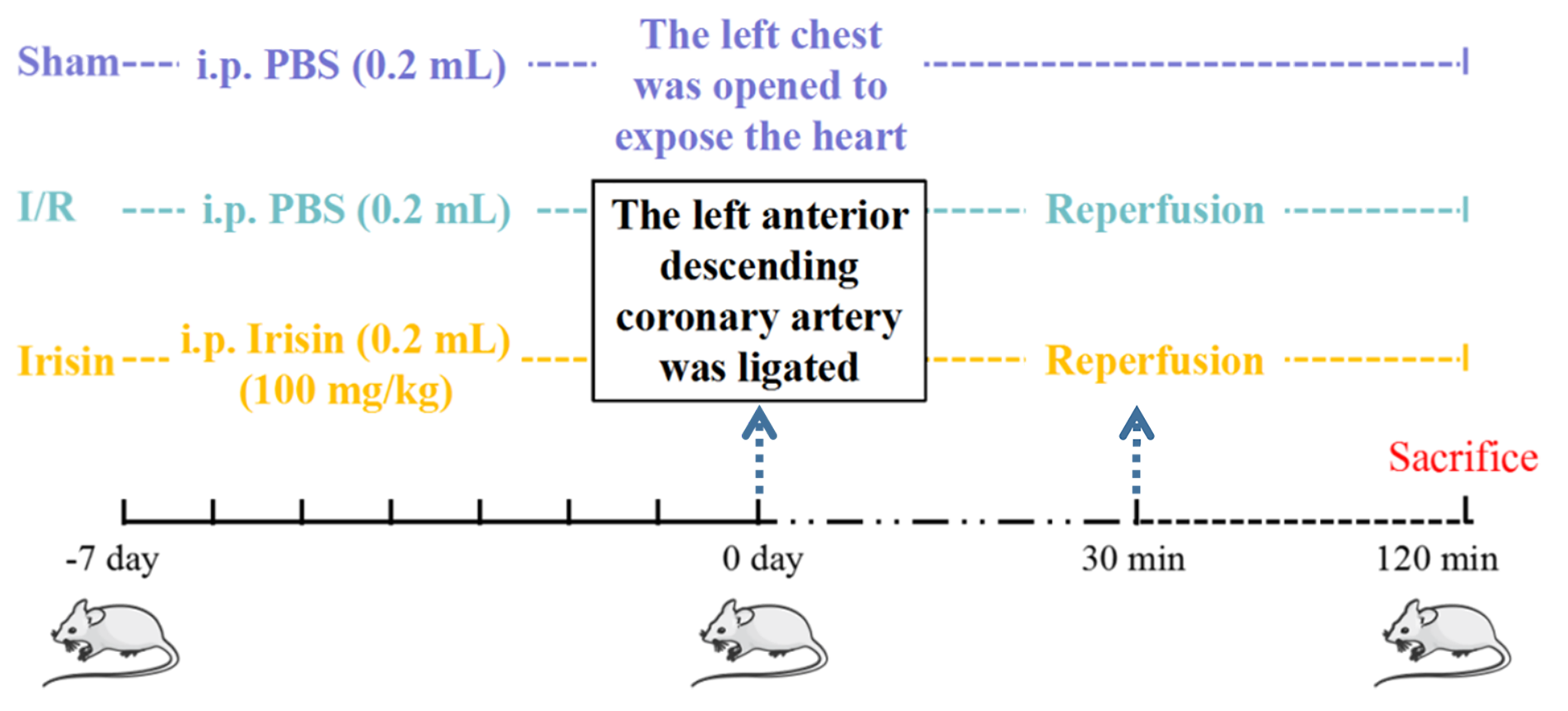

Supplement: S1 Fig — Timeline of the experimental process of this research treatment and ischemia-reperfusion (I/R) injury induction in rats. (i. p.: intraperitoneal injection). (TIF) [file pone.0291022.s001.tif]

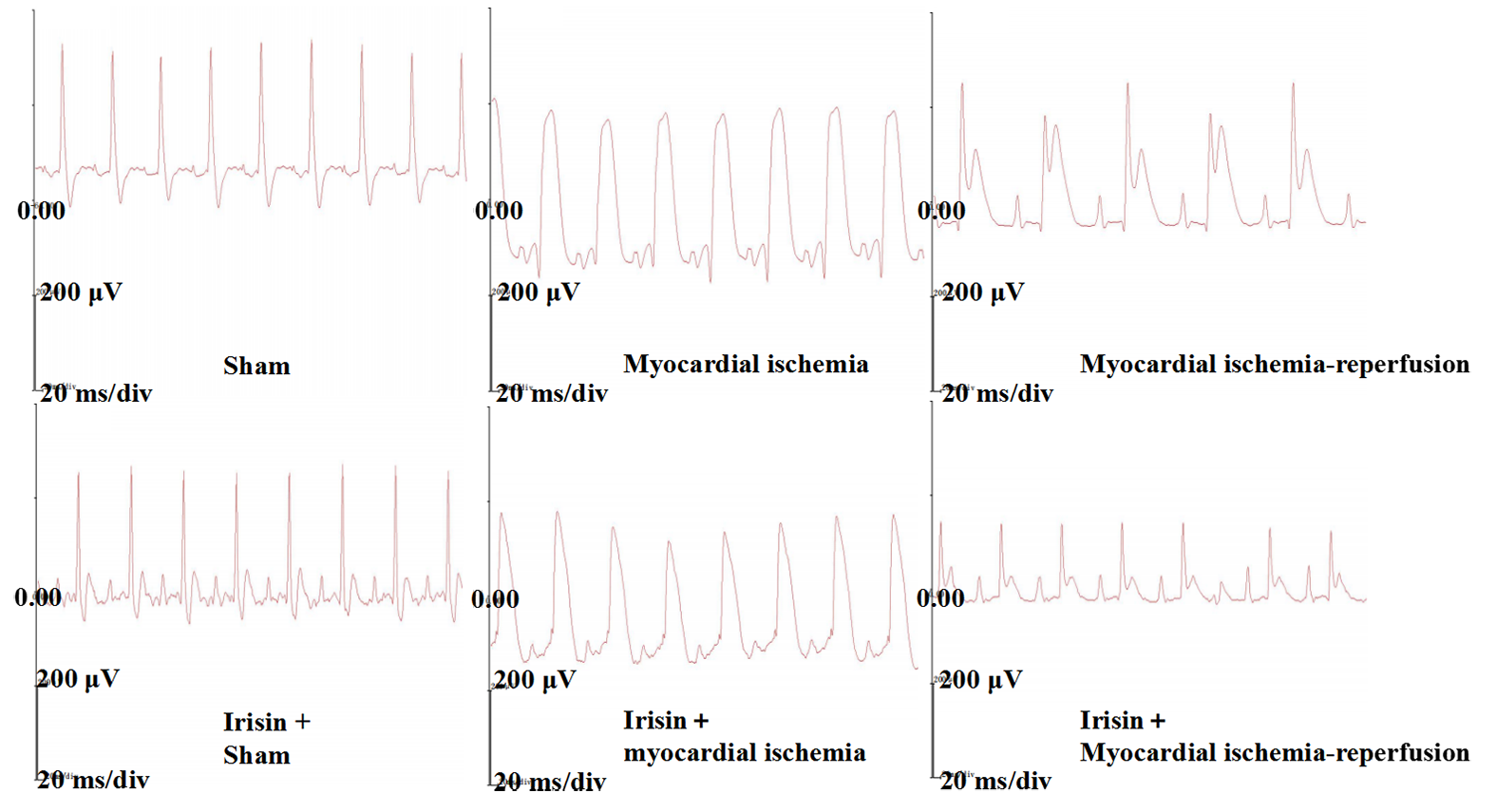

Supplement: S2 Fig — (TIF) [file pone.0291022.s002.tif]

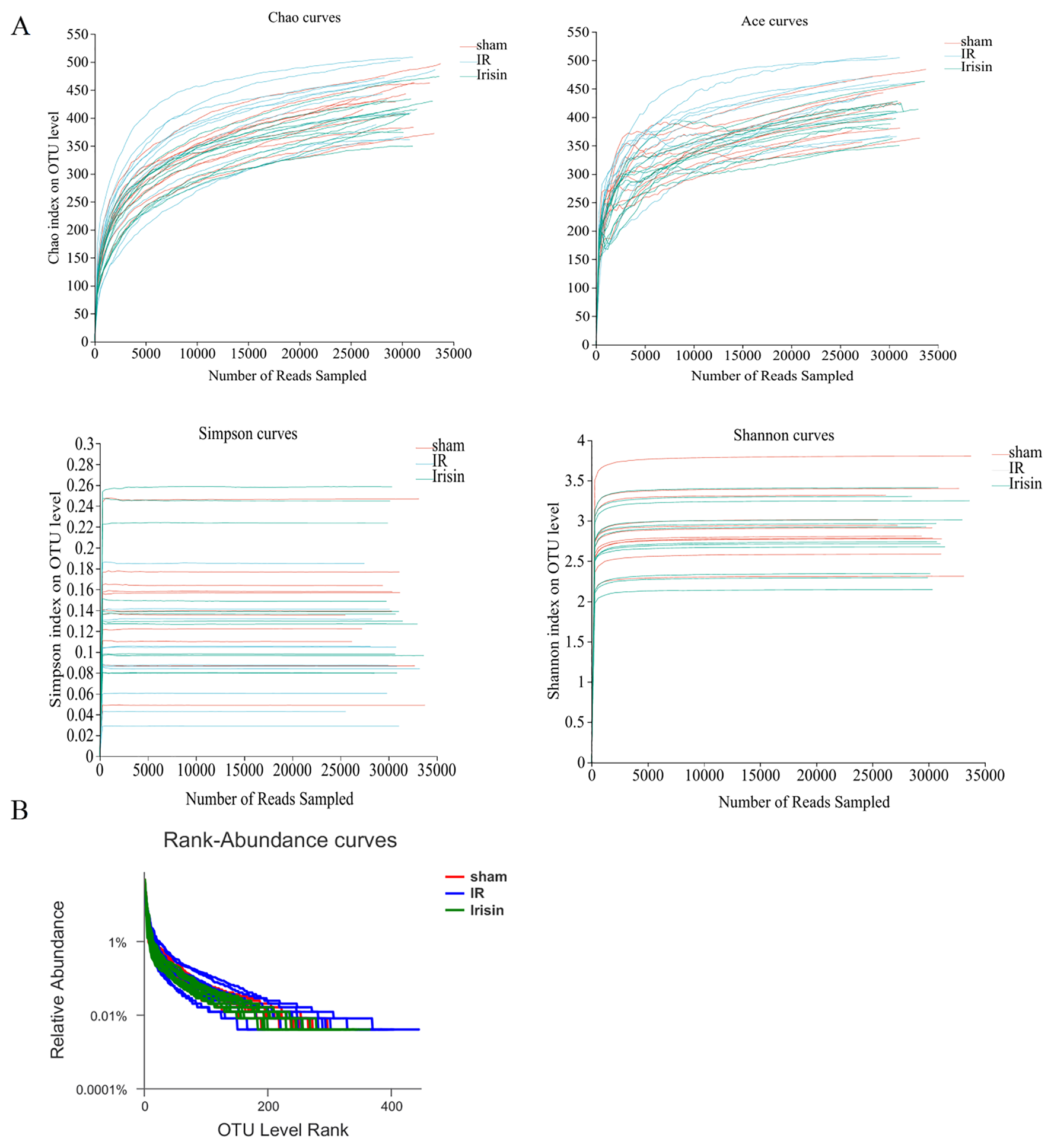

Supplement: S3 Fig — (A) Rarefaction curve indicating that the amount of sequencing reads per sample has reached saturation. (B) Rank-Abundance curves were shown. (TIF) [file pone.0291022.s003.tif]

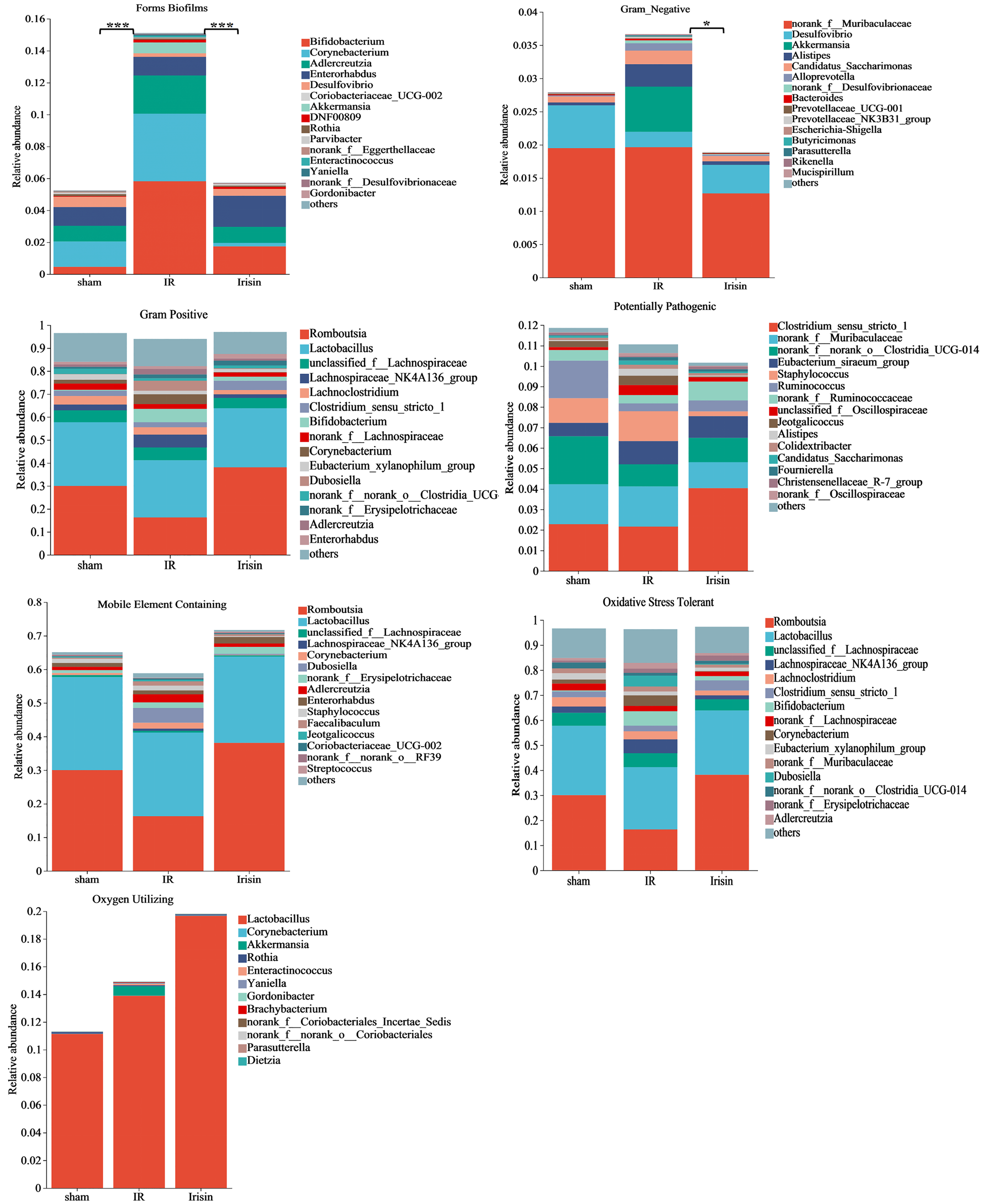

Supplement: S4 Fig — The relative abundance of Biofilm Forming, Gram Positive, Gram Negative, Pathogenic Potential, Mobile Element Containing, Oxidative Stress Tolerant and Oxygen Utilizing were shown. (TIF) [file pone.0291022.s004.tif]
